# A Rare Case of Asymptomatic Massive Colonic Lithobezoar in a Young Child

**DOI:** 10.7759/cureus.29538

**Published:** 2022-09-24

**Authors:** Qaidar Alizai, Farhan Ullah, Jamshed Alam, Umi Aiman, Tabish Ahmad

**Affiliations:** 1 Department of General Surgery, Hayatabad Medical Complex, Peshawar, PAK; 2 Department of Internal Medicine and Pediatrics, The MetroHealth System, Cleveland, USA; 3 Department of Radiology, Mian Rashid Hussain Shaheed Memorial Hospital, Pabbi, PAK

**Keywords:** colonic bezoar, asymptomatic colonic bezoar, colonic lithobezoar, pica, lithobezoar, bezoar, stone-bezoar

## Abstract

Colonic bezoar is a rare condition of accumulation of foreign bodies or non-nutritious material in the large intestine, usually presenting with symptoms of obstruction. Colonic lithobezoar is an even more rare type of condition with only 12 cases reported in the literature to date. We present a case of a young, intellectually disabled kid, who was diagnosed incidentally with lithobezoar after a road traffic accident. The first-line treatment for uncomplicated non-obstructed bezoar is a medical treatment with laxatives and fluids. For acutely obstructed bezoars, the treatment of choice is evacuation under general anesthesia. Surgical evacuation may be considered a last resort in complicated or refractory cases. Moreover, regardless of obstruction, all cases must be treated as inpatients and must receive a psychiatric and hematologic evaluation.

## Introduction

Bezoar is the term used for the accumulation of indigestible material in the gastrointestinal tract, whether food particles or foreign bodies [1]. The most common location for accumulation is the stomach; however, these substances may rarely accumulate in the intestine, rarely causing colonic bezoar. Lithobezoar means the accumulation of pebbles or pieces of bricks, stones, or related items in the gut. Colonic lithobezoar means accumulation of stones in the large intestine, which is an even rare condition that usually presents with the signs and symptoms of intestinal obstruction [2].

## Case presentation

**Figure 1:**
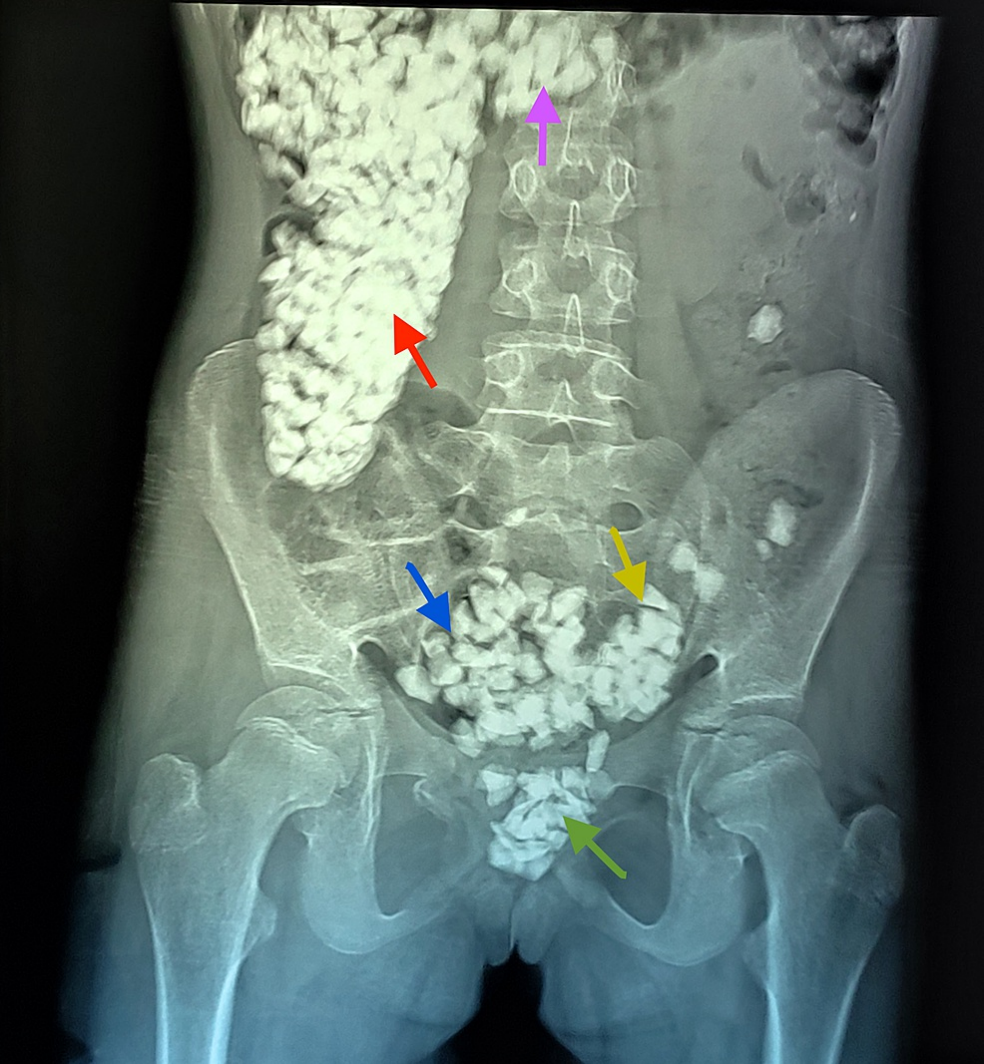
First abdominal X-ray of the patient taken in the emergency department. Plain abdominal radiograph (frontal projection) showing multiple radiopaque densities of variable sizes in the ascending (red arrow), proximal transverse (purple arrow), distal descending (yellow arrow), and sigmoid colon (blue arrow) along with the rectum (green arrow).

We present this interesting case of a 14-year-old female child with mental and developmental delay, who was incidentally diagnosed with massive colonic bezoar. The patient was brought to the emergency department of Hayatabad Medical Complex after a road traffic accident (pedestrian vs. vehicle) in September 2021. She was clinically stable during the primary and secondary surveys. She was complaining of mild bruises and pain in the right upper and lower limbs. To rule out any abdominal trauma, an abdominal X-ray was done, which revealed multiple irregular radiopaque shadows with the typical "corn on the cob" appearance extending from the cecum to the rectum. Figure 1 shows the initial plain abdominal X-ray.

**Figure 2:**
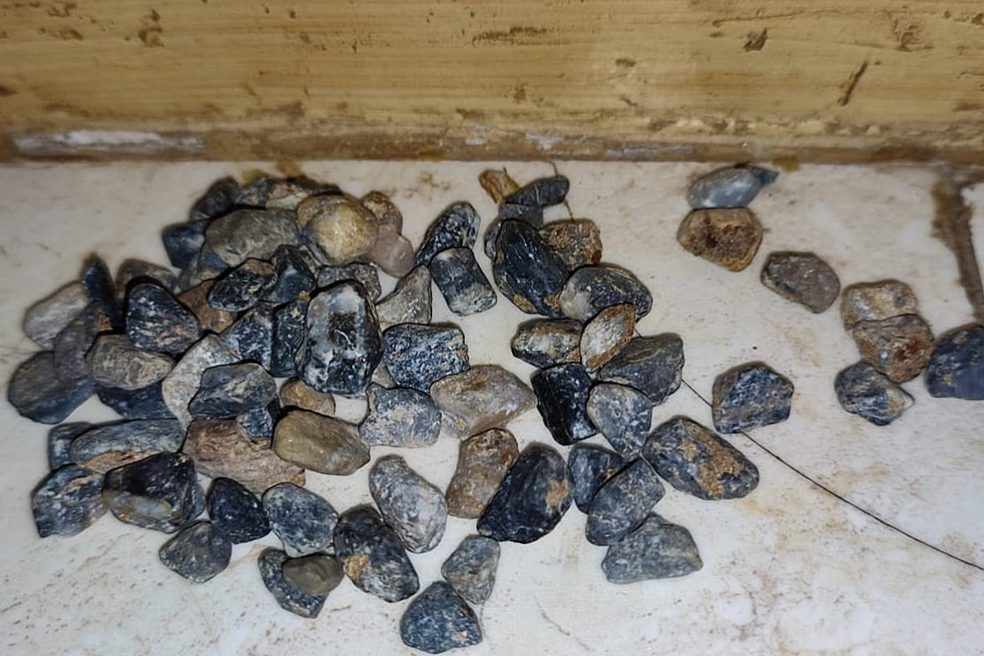
Image showing the spontaneously passed lithobezoar.

After her trauma evaluation was complete, further history was elicited, which revealed that she had been seen eating stones (pica) multiple times, by her community members, for the past seven years. Since she remained asymptomatic, no medical care was sought. On examination, we found a moderately pale-looking child with abnormal facial features, expressionless face, polydactyly, tachycardia, and an ill-defined heart murmur, suggestive of congenital disorders (differential diagnoses were Patau's syndrome and Diamond-Blackfan syndrome, but no formal diagnosis was made). Abdominal examination showed mild tenderness on deep palpation and slightly hyperactive bowel sounds. A digital rectal examination was not performed due to the patient's irritability. We immediately admitted the patient to the surgery ward for conservative management. She was started on laxatives and parenteral fluids, which helped her pass the stones in stools daily. Figure 2 shows the spontaneously passed lithobezoar.

**Figure 3:**
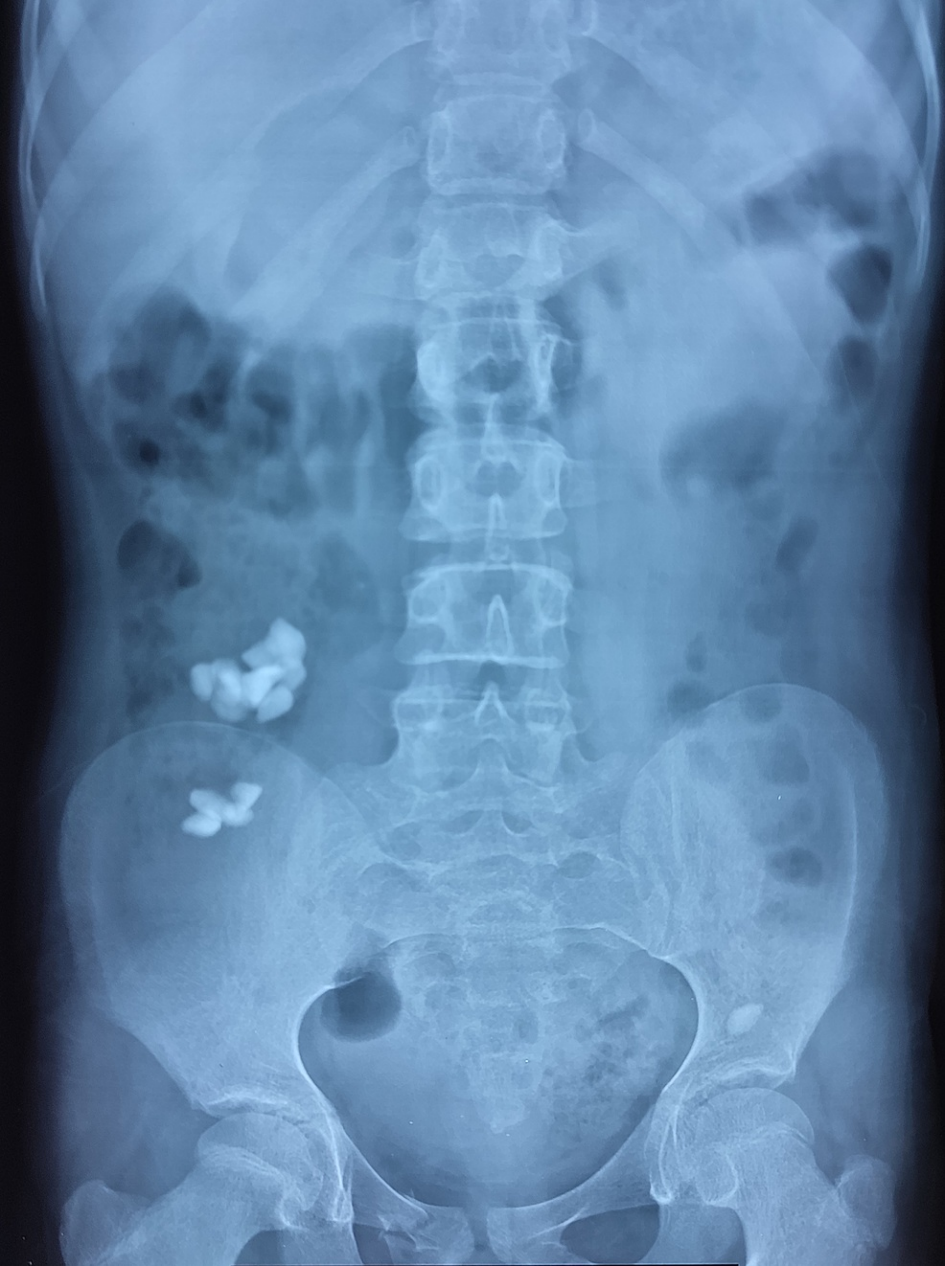
Plain abdominal X-ray on follow-up visit after a week. Follow-up plain abdominal radiograph (frontal projection) showed significant improvement in the disease process that was noted in the previous old X-rays.

Her blood investigations (Table 1) suggested microcytic anemia. Her condition improved significantly in a few days; hence, she was discharged home on laxatives, multivitamins, and iron supplements on the fourth day of admission, with a daily follow-up plan with serial X-rays for the next few days. On her one-week follow-up visit to the clinic, she was more active and smiling. There was a great deal of improvement noticed in her clinical status. The follow-up X-ray of the abdomen (Figure 3) revealed partial clearance of the lithobezoar. Laxatives were continued to ensure complete evacuation. She was referred to pediatric psychiatry and genetics for further evaluation. On a two-month follow-up visit, blood investigations showed improvement in the hemoglobin and iron levels and with that, the pica behavior was resolved.

**Table 1:** Details of the initial blood workup of our patient.

	Level	Unit	Normal range
Hemoglobin (Hb)	10.9	g/dl	11.5-17.5
Hematocrit (Hct)	35.5	%	36-54
Mean corpuscular volume (MCV)	58.6	Fl	76-96
Mean corpuscular hemoglobin (MCH)	18	pg	27-33
Mean corpuscular hemoglobin concentration (MCHC)	30.7	g/dL	33-35
Red cell distribution width (RDW)	9.81	%	11.5-33.5
Total leukocyte count (TLC)	14,700	per μL	4,000-11,000
Platelets	223,000	per μL	150-450
Serum iron	98.4	μg/dl	50-170
Total iron-binding capacity (TIBC)	250.6	μg/dl	250-400
Saturation of transferrin	39.2	%	15-50
Serum ferritin	27.7	ng/ml	14-150
Serum phosphate	1.3	mmol/L	0.8-1.5
Serum magnesium	2.1	mg/Dl	1.7-2.55

## Discussion

Bezoar can be of many types. The name of bezoar is based on the constituent material, more commonly including the trichobezoar (hair), phytobezoar (vegetable and fruit particles), lactobezoar (milk and curd accumulation), and rare items like metal (metal bezoar) or stones (lithobezoar) [3]. The most common risk factors associated with bezoar are pica, behavioral disturbances in young girls, psychiatric illness, intellectual disability, low socioeconomic status, parental neglect in young children, and poor educational status. Pica is the condition of consuming non-nutritious substances, which itself is associated with iron deficiency, pregnancy, infancy, and other conditions [4]. These non-nutritious substances usually accumulate in the stomach and block the pyloric sphincter, presenting with feeding intolerance, early satiety, vomiting, and abdominal pain. However, rarely, the material accumulates in the colon, causing colonic bezoar, which then presents with constipation, straining on defecation, painful defecation, vomiting, and abdominal pain. On examination, they may have signs of abdominal obstruction like tender, distended abdomen, palpable hard masses per abdomen, and the typical “colonic crunch” sign per rectal examination. Colonic lithobezoar is specifically rare, with only 12 cases reported in the literature so far [1-11]. Table 2 illustrates the summary of these studies. Our case will be the 13th of the kind. Unlike the previous cases, which presented with symptoms of obstruction, the bezoar in our patient came to notice incidentally when she was brought to the hospital after a road traffic accident (RTA).

**Table 2:** The detailed summary of all the colonic lithobezoar cases reported in the literature till 2021. M: male; F: female; RTA: road traffic accident; DRE: digital rectal examination: LIF: lower iliac fossa; Hb: hemoglobin; Hct: hematocrit; GA: general anesthesia.

S. no	Study	Age (years)	Sex	Chief complaints	Physical examination	imaging	Labs				Pica	Mental development	Treatment
							Hb (g/dl)	Hct (%)	Iron (μg dl)	Ferritin (ng/ml)			
1	Current case	14	F	RTA, incidental diagnosis of lithobezoar	Normal abdominal examination	X-ray	10.9	35.5	98.4	27.7	+	Intellectually disabled	Laxatives and oral fluids
2	Aihole [2]	7	M	Irregular bowel habits for 6 months	Hard masses palpable per abdomen, DRE revealed hard stones	X-ray	7.2	-	-	-	+	Normal	Rectal enema for 3 days
3	Aihole [2]	5	M	Pain abdomen, diarrhea, passing stones in stools	Tender left flank, hard colonic stools palpable	X-ray	7.6	-	-	-	+	Normal	Rectal enemas for 2 days
4	Senol et al. [4]	7	F	Abdominal pain, constipation	Tender, distended abdomen, hard fecaloid masses on DRE, colonic crunch sign	X-ray	6.8	22.5	14	9	+	Normal	Evacuation under GA
5	Oner et al. [1]	14	M	Constipation, abdominal pain	Pallor, mild LIF tenderness, reduced bowel sounds, generalized abdominal tenderness	X-ray	5.5	19	6	1	+	Normal	Evacuation under GA
6	Sheikh et al. [3]	9	M	Recurrent abdominal pain and constipation, failure to thrive for 3 years	Abdominal distension, palpable hard masses, stone pellets protruding through the anus	X-ray	N/A	N/A	N/A	N/A	+	Normal	Evacuation under GA
7	Mohammad [8]	8	M	Constipation, abdominal pain, painful defecation	Palpable hard masses per abdomen, stone pellets on DRE	X-ray	N/A	N/A	N/A	N/A	+	Normal	Evacuation under GA
8	Numanoğlu and Tatli [5]	4	M	Abdominal distension, constipation	Abdominal distension, hyperactive bowel sounds, multiple irregular masses on abdominal palpation, hard masses on DRE, colonic crunch sign	X-ray	6	21.1	N/A	N/A	+	Normal	Evacuation under GA
9	Narayanan et al. [7]	9	M	Constipation, abdominal pain, painful defecation	Palpable hard masses per abdomen, hard masses, and blood stains on DRE	X-ray	N/A	N/A	N/A	N/A	+	Mild retardation	Evacuation under GA
10	Tokar et al. [6]	6	F	Constipation, recurrent abdominal pain, painful defecation	Hard masses palpable per abdomen, hard pellets on DRE	X-ray	N/A	N/A	N/A	N/A	+	Normal	Evacuation under GA
11	Vijayambika [9]	6	M	Constipation, abdominal pain	Pallor, mild LIF tenderness	X-ray	N/A	N/A	N/A	N/A	+	Normal	Laxatives and oral fluids
12	Ratan and Grover [10]	5	F	Constipation	N/A	X-ray	N/A	N/A	N/A	N/A	N/A	Intellectually disabled	Evacuation under GA
13	Rathi and Rathi [11]	4	F	Constipation, abdominal pain, painful defecation	N/A	X-ray	N/A	N/A	N/A	N/A	N/A	Normal	Evacuation under GA

Almost all the cases in the literature are associated with pica, and most of them have iron deficiency anemia. However, only two of the 13 cases reported intellectual disability. Most of the reported cases are from India (seven out of 12) and Turkey (four out of 12). The management in most of these cases was the digital evacuation of the stones under general anesthesia (GA) to relieve the acute obstruction. In contrast, our case was treated conservatively with laxatives and fluids only, as there was no acute obstruction. Most surgeons prefer the former method, as it is safe, quick, and pain-free. Though, in otherwise stable and cooperative patients, one may choose to opt for only conservative management, like colonic lavage and laxatives. Surgical evacuation should be the last line option, only for refractory or complicated cases, if laxatives and manual or colonoscopic decompressions fail. Also important is the fact that in cases of acute obstruction, the surgical evacuation should be done on an emergent basis to avoid a closed loop obstruction of the large bowel between the bezoar and the ileocecal valve.

## Conclusions

It is concluded that lithobezoar is a rare condition that should be excluded if a young child presents with abdominal pain or bowel obstruction. Moreover, primary care physicians should evaluate intellectually disabled kids for pica and bezoars during wellness visits. Regardless of presentation, whether incidental or obstruction, colonic bezoar patients must be treated as inpatients with oral laxatives or enema to help with the spontaneous evacuation of the lithobezoar, followed by regular follow-up visits with serial X-rays to prevent any complications and recurrence of the condition. If conservative management fails, then manual evacuation should be done as a second-line treatment. Furthermore, all the patients must receive a psychiatric, genetic, and hematologic evaluation. Occupational and physical therapy assessments should be requested. Finally, any associated iron deficiency must be treated with oral or parenteral supplements.

## References

[1] Oner N, Kaya Z, Turkyilmaz Z, Karabulut R (2012). An unusual cause of iron deficiency anemia in a child with colonic lithobezoar: case report and review of the literature. J Hematol.

[2] Aihole J (2018). Colonic lithobezoar: our experience in children. J Paediatr Child Health.

[3] Sheikh MS, Hilal RM, Misbha AM, Farooq AR (2010). Colorectal lithobezoar: a rare case report. J Indian Assoc Pediatr Surg.

[4] Senol M, Ozdemir ZÜ, Sahiner IT, Ozdemir H (2013). Intestinal obstruction due to colonic lithobezoar: a case report and a review of the literature. Case Rep Pediatr.

[5] Numanoğlu KV, Tatli D (2008). A rare cause of partial intestinal obstruction in a child: colonic lithobezoar. Emerg Med J.

[6] Tokar B, Ozkan R, Ozel A, Koku N (2004). Giant rectosigmoid lithobezoar in a child: four significant clues obtained from history, abdominal palpation, rectal examination and plain abdominal X-ray. Eur J Radiol.

[7] Narayanan SK, Akbar Sherif VS, Babu PR, Nandakumar TK (2008). Intestinal obstruction secondary to a colonic lithobezoar. J Pediatr Surg.

[8] Mohammad MA (2010). Rectosigmoid lithobezoar in a eight-year-old. Afr J Paediatr Surg.

[9] Vijayambika K (2004). Lithobezoar. Indian Pediatr.

[10] Ratan SK, Grover SB (2000). Giant rectosigmoid stone bezoar in a child. Clin Pediatr (Phila).

[11] Rathi P, Rathi V (1999). Colonic lithobezoar. Indian J Gastroenterol.

